# EGFP oligomers as natural fluorescence and hydrodynamic standards

**DOI:** 10.1038/srep33022

**Published:** 2016-09-13

**Authors:** György Vámosi, Norbert Mücke, Gabriele Müller, Jan Wolfgang Krieger, Ute Curth, Jörg Langowski, Katalin Tóth

**Affiliations:** 1 Department of Biophysics and Cell Biology, Faculty of Medicine, University of Debrecen, H-4032 Debrecen, Hungary; 2 DKFZ, Biophysics of Macromolecules, D-69120 Heidelberg, Germany; 3 Institute for Biophysical Chemistry, Hannover Medical School, D-30625 Hannover, Germany

## Abstract

EGFP oligomers are convenient standards for experiments on fluorescent protein-tagged biomolecules. In this study, we characterized their hydrodynamic and fluorescence properties. Diffusion coefficients D of EGFP_1–4_ were determined by analytical ultracentrifugation with fluorescence detection and by fluorescence correlation spectroscopy (FCS), yielding 83.4…48.2 μm^2^/s and 97.3…54.8 μm^2^/s from monomer to tetramer. A “barrels standing in a row” model agreed best with the sedimentation data. Oligomerization red-shifted EGFP emission spectra without any shift in absorption. Fluorescence anisotropy decreased, indicating homoFRET between the subunits. Fluorescence lifetime decreased only slightly (4%) indicating insignificant quenching by FRET to subunits in non-emitting states. FCS-measured D, particle number and molecular brightness depended on dark states and light-induced processes in distinct subunits, resulting in a dependence on illumination power different for monomers and oligomers. Since subunits may be in “on” (bright) or “off” (dark) states, FCS-determined apparent brightness is not proportional to that of the monomer. From its dependence on the number of subunits, the probability of the “on” state for a subunit was determined to be 96% at pH 8 and 77% at pH 6.38, i.e., protonation increases the dark state. These fluorescence properties of EGFP oligomeric standards can assist interpreting results from oligomerized EGFP fusion proteins of biological interest.

Protein complexes in live cells are often investigated by observing fluorescent protein (FP) tags. Intracellular concentration, aggregation state and mobility of the labeled protein of interest can be quantified by fluorescence microscopic tools: fluorescence correlation spectroscopy (FCS), number and brightness analysis (N&B), Förster resonance energy transfer (FRET), fluorescence recovery after photobleaching (FRAP), etc.^1^,^2^,^3^,^4^,^5^,^6^. Proteins can be present in various oligomerization states, which may differ in their mobility and brightness. The measured values of these parameters are influenced by the photophysical behavior of the FP, which depends on the measurement conditions (excitation light intensity, duration of illumination, pH, etc.). Different photophysical processes of individual EGFP and other fluorescent proteins have already been described (photobleaching, triplet state formation, protonation- or light-induced “blinking”)^7^,^8^,^9^,^10^,^11^,^12^,^13^. There are still open questions how these processes contort the measured parameters (apparent brightness, apparent diffusion coefficient) of protein complexes containing more than one fluorescent protein. Earlier Pack and coworkers^14^ and Dross in our lab^5^ reported FCS-derived diffusion coefficients of EGFP oligomers in live cells and crude cell lysates. Here we characterize the fluorescence and hydrodynamic properties of recombinant covalently linked EGFP oligomers composed of 1 to 4 EGFP subunits in solution in order to validate them as natural standards for higher protein complexes. We test the effect of illumination power on the fluorescence parameters of the EGFP oligomers at two pH values and interpret how they relate to the behavior of the constituting monomeric subunits. We compare FCS-derived diffusion coefficients of EGFP oligomers with values yielded by analytical ultracentrifugation. Finally we propose a simple procedure to quantify the probability of long-lived dark states of EGFP by analyzing the apparent brightness of the oligomers as a function of the number of subunits.

## Results

### Characterization of EGFP oligomers by analytical ultracentrifugation

To use EGFP oligomers of 1 to 4 covalently linked subunits as calibration standards, we determined their molar masses and hydrodynamic parameters in solution by analytical ultracentrifugation with fluorescence detection system (AU-FDS). Sedimentation equilibrium runs were recorded at several speeds and protein concentrations. Molar masses were determined by globally fitting the radial distribution of the fluorescence signals at all rotor speeds for each oligomer with a single species model (Supplementary Fig. S1) using the software BPCFIT^15^. For EGFP_1–3_ the molar masses, *M*_*eq*_, (Table 1) agreed within 5% with the sequence-based calculation *M*_*calc*_ of the EGFP monomer (26.9 kg/mol) and its multiples. Our *M*_*eq*_ value for the EGFP monomer agrees well with that reported earlier using a similar setup^16^,^17^. For EGFP_4_ the *M*_*eq*_ value was 10% smaller than *M*_*calc*_. This deviation and the systematic divergence of the fit residuals observed for EGFP_4_ (Supplementary Fig. S1) indicate an additional pool of smaller particles. Indeed, some minor faster, fluorescent species were also present in the lanes of EGFP_4_ and also EGFP_1_ in the native polyacrylamide gel (Supplementary Fig. S2B), which may be degradation products or, in the case of the tetramer, a lower oligomer. The amount of neither impurity exceeds 5% in these preparations.

**Figure 1:**
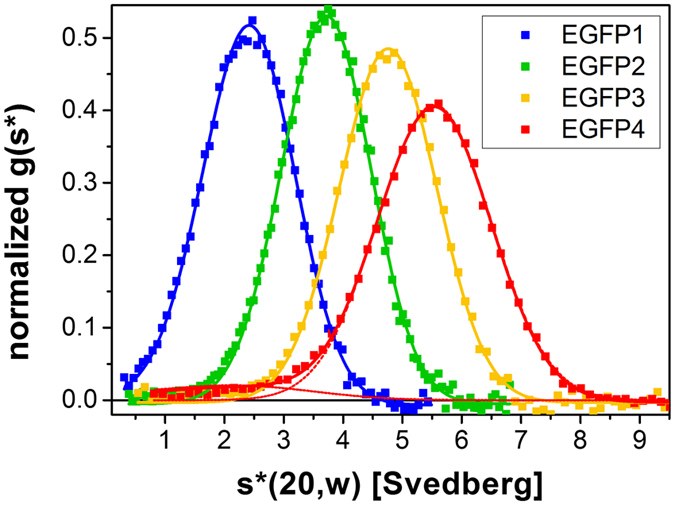
Sedimentation velocity centrifugation of the EGFP oligomers with fluorescence detection. Normalized representative g(s*) plots are displayed. Sedimentation coefficients were converted to 20 °C and water as solvent. Distributions were fitted with a single Gaussian peak for EGFP_1–3_ and with two Gaussians for EGFP_4_; the second minor component (5%) present in the EGFP_4_ sample is shown at a lower s* value. The peaks yielded s_vel_, and peak broadening D_vel_ values.

The homogeneity of the EGFP oligomers in size and shape was then characterized by sedimentation velocity runs at 50,000 rpm using four different protein concentrations between 50 and 400 nM. Figure 1 shows the sedimentation velocity analysis at 400 nM EGFP subunits. The normalized distributions of the sedimentation coefficients for EGFP_1–3_ could be fitted with a single Gaussian peak, while EGFP_4_ had a second minor component (5%) in the lower *s* value range as seen in the equilibrium runs and in the gel. The single peaks suggest that each oligomer has a unique arrangement of the subunits, which may be due to the short length of the linkers limiting the possible conformations. *s*_*vel*_ values of the main peaks are listed in Table 1. Diffusion coefficients, *D*_*vel*_, were assessed from peak broadening^18^,^19^ (Table 1). Molar masses, *M*_*vel*_, were determined from *s*_*vel*_ and *D*_*vel*_ values (Table 1). The excellent agreement between the *M*_*vel*_, *M*_*eq*_ and *M*_*calc*_ values for EGFP_1–3_ indicates that these samples were highly homogeneous.

**Figure 2:**
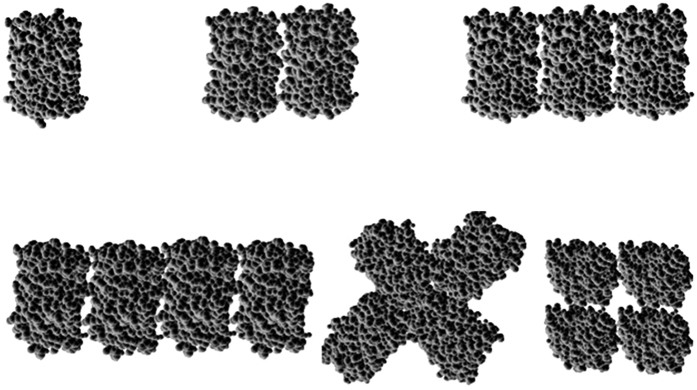
Possible spatial arrangements of the oligomers based on the crystal structure of EGFP. Spatial restrictions are imposed by the 5 amino acid linkers between the subunits.

The possible shape of the oligomers may also be inferred from the sedimentation coefficients. The monomeric subunits can assume various spatial arrangements in the oligomers. We calculated a sedimentation coefficient *s*_*calc*_ = 2.6 S for monomeric EGFP based on the crystal structure with the HYDROPRO software, which agreed well with the measured *s* value of 2.52 S. For the oligomers, the sedimentation coefficients of some possible spatial arrangements (Fig. 2) were also estimated by this software. Here, a constraint was taken into account: both the C and N-termini of a barrel-shaped monomer are on the top of the barrel^20^, and two neighboring barrels are connected by 5-amino-acid linkers. In the case of a side-by-side linear arrangement the deviation of *s*_*calc*_ from the measured *s*_*vel*_ values was between 2–5% for all the oligomers (Table 1). A star- or square-shaped conformation of the tetramer yielded larger deviations between the model-based calculated *s* value and and the measured *s* values (9 and 11%, respectively). Thus, the oligomers are probably arranged in an elongated rather than a compact shape. Pack *et al*.^14^. also suggested an elongated rather than spherical shape for EGFP_2–5_ oligomers connected with 25 aa long linkers, much longer than our 5 aa linkers.

### Effect of oligomerization on the fluorescence properties of EGFP

We tested the influence of EGFP oligomerization on the absorption and fluorescence emission spectra, anisotropy and lifetime. Normalized absorption spectra overlapped perfectly, but emission spectra were red-shifted by ~2 nm from monomer to tetramer (Supplementary Fig. S3). This slight bathochromic shift may be due to direct long-range interaction between EGFP fluorophores. Fluorescence lifetime decay curves could be well fitted to single exponentials. Lifetimes decreased monotonously from the monomer to the tetramer by 4% (Table 2).

This suggests that no or only little energy transfer takes place from fluorescent to non-emitting dark subunits in an oligomer. The steady-state fluorescence anisotropy decreased progressively from 0.32 for the monomer to 0.23 for the tetramer, which provides evidence for homo-FRET between the subunits^21^,^22^.

As shown, the bulk fluorescence parameters: the emission peak wavelength, lifetime and anisotropy changed progressively with the size of the oligomer. Next, we investigated the fluorescence properties of individual oligomers by using fluorescence correlation spectroscopy.

### Fluorescence correlation spectroscopy of EGFP oligomers

The state of aggregation of a fluorescently tagged protein can be assessed from the diffusion coefficient and the molecular brightness. However, the measured values of these parameters are influenced by the photophysical behavior of the fluorophore, which in turn depends on the experimental conditions (excitation intensity, pH, etc.). In our fluorescence correlation spectroscopy (FCS) experiments, we varied the illumination power of the laser and the pH of the buffer solution, and recorded fluorescence intensity and autocorrelation functions (ACFs). ACFs were fitted to obtain the apparent diffusion coefficients, concentrations and molecular brightness values for each oligomer. With this model system we could show how to estimate the “real” parameters from the measured apparent values.

#### Fluorescence intensity

**Figure 3:**
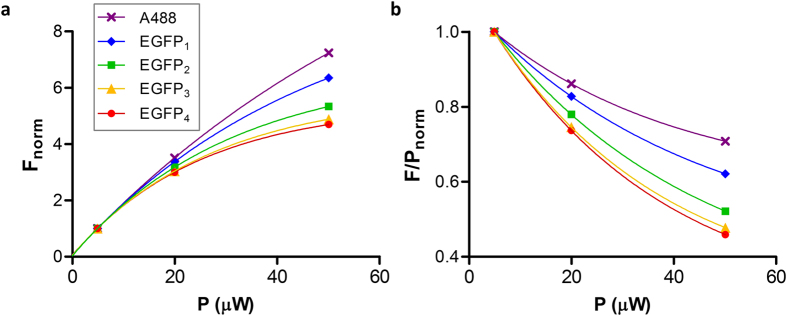
Dependence of the relative fluorescence intensities of EGFP_1–4_ and Alexa 488 on the illumination power. (**a**) Raw fluorescence intesities normalized to 1 at the lowest illumination power. (**b**) Fluorescence intensities divided by the illumination power and normalized as above. Fits are single exponentials. Measurements were done in 20 mM Tris-HCl, 100 mM NaCl, pH 8.

First we analyzed the FCS-derived average fluorescence intensities, F, of the oligomers as a function of the illumination power, P (Fig. 3). Measurements were done in Tris buffer, pH 8 at 1, 4 and 10% laser power corresponding to 5, 20 and 50 μW at the microscope objective. As a comparison, the behavior of the Alexa Fluor 488 dye (A488) is also shown. Fluorescence intensities (normalized at 5 μW) increased with increasing laser power (Fig. 3a), but not linearly and not uniformly for the different molecules. The highest rate of increase was observed for A488, followed by EGFP_1_ and got less as the size of the oligomer increased. To visualize the deviation from linearity, F was divided by the illumination power and normalized to 1 at 5 μW (Fig. 3b). The decreasing tendency of the graphs means that at higher illumination power fewer photons were emitted per incoming photons. The decrease became greater with increasing oligomer size. An obvious reason for this effect is a light-induced transition to a dark state, i.e., photobleaching or blinking, which becomes more prominent for larger molecules moving more slowly and residing longer in the focal volume. This means that a simple intensity measurement with strong illumination may lead to an underestimation of the concentration of fluorescent molecules to an extent depending on the mobility and the laser power.

#### ACF amplitude

**Figure 4:**
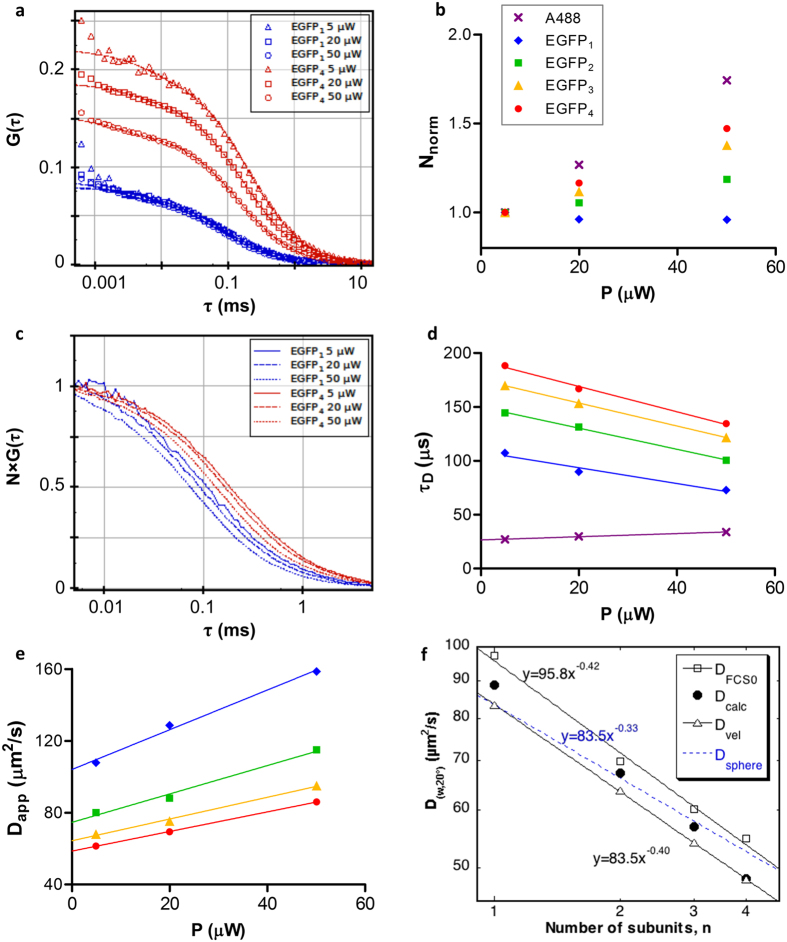
Analysis of autocorrelation curves and their parameters as a function of oligomer size and illumination power. (**a**) Representative autocorrelation functions for EGFP1 and EGFP4 at different illumination powers at pH 8. (**b**) Normalized particle numbers obtained from fits of the ACFs of EGFP_1–4_. Alexa 488 is shown as a comparison. (**c**) Normalized representative ACF curves from part a). (**d**) Diffusion times obtained from fitting ACFs of EGFP_1–4_ and Alexa 488. (**e**) Apparent diffusion coefficients of EGFP_1–4_ calculated from the diffusion times according to equation (9). Intercepts of linear fits define the zero light extrapolated D_FCS0_ values. (**f**) Comparison of diffusion coefficients obtained by different methods. D_calc_ was calculated from s_calc_ with the Svedberg equation, assuming side-by-side linear arrangement of barrels (see Fig. 2). D_vel_ was obtained from peak broadening of sedimentation velocity runs (see Fig. 1). D_FCS0_ values were extrapolated from FCS-derived D values to zero light conditions. Experimental D values vs. n were fitted to power functions yielding similar powers. D dependence for a sphere is given as comparison.

A principally different method to determine the concentrations from the same data is the analysis of autocorrelation functions, which in addition yields the diffusion coefficients. As an example, Fig. 4a shows the ACF curves of EGFP_1_ and EGFP_4_ at different illumination powers (curves for Alexa 488, EGFP_2_ and EGFP_3_ are not shown). ACFs were fitted to a model assuming triplet state formation and a single free diffusion component. The fast decay at the beginning of the curve (in the μs range) is related to triplet state formation. The fit yields *N*, the average number of molecules in the focal volume (the reciprocal of the amplitude) and the diffusion time (*τ*_*D*_, the mean dwell time) or the related diffusion coefficient, *D* (see Materials and Methods). With increasing illumination power the amplitudes of ACF curves decreased for EGFP_2–4_ and for A488, but remained constant (or decreased slightly) for the EGFP_1_. Accordingly, the apparent normalized particle numbers increased: Fig. 4b shows an approximately linear relationship of *N*_*norm*_ with the laser power. The slope increased with size of the oligomers. With increasing illumination power, excitation at the center of the illuminated area may be saturated because EGFP cannot absorb another photon during its nanosecond fluorescence lifetime. This flattens the detection efficiency profile and widens the observation volume, i.e., photons from an area of larger radius will contribute to the signal^23^. This may lead to an increase of the apparent particle number. On the other hand, the apparent particle number may also decrease due to photobleaching with increasing illumination power. These two effects were separately simulated for EGFP_1–4_ by using Monte-Carlo method, and resulted in the expected dependence of the apparent particle number on the illumination power (Supplementary Fig. S4a for saturation and Supplementary Fig. S4b for photobleaching). In these simulations the different oligomers behaved similarly, contrary to the divergence seen in the measurements (Fig. 4b). This discrepancy suggests that further photophysical processes could play a role. In our measurements saturation seemed to dominate over bleaching for EGFP_2–4_, whereas for EGFP_1_ these effects canceled each other. The A488 dye is very photostable, its bleaching was not significant at these laser powers; therefore, saturation dominated and the fitted *N* value increased with the laser power. These effects must be taken into account for exact concentration measurements by FCS to avoid significant errors (here, ~50% error in *N* would occur at a 10 fold variation of the laser power).

#### Diffusion time and diffusion coefficient

The other principal parameter of the ACF is the correlation time *τ*_*D*_. To analyze its behavior at different illumination powers, we normalized the curves at the plateau amplitudes to 1, and presented the time interval of interest for EGFP_1_ and EGFP_4_ as examples in Fig. 4c (EGFP_2_ and EGFP_3_ behaved similarly and the curves lied between those of the EGFP_1_ and EGFP_4_). EGFP_1–4_ curves were all shifted to the left, decaying at shorter times, contrary to Alexa 488 curves, which were shifted to the right with increasing illumination power^24^. Apparent diffusion times (*τ*_*D*_) are shown in Fig. 4d. The apparent increase of *τ*_*D*_ for A488 probably stems from the same saturation effect as the apparent increase of its *N*: the observation volume for this dye is increased and therefore the dwell time is longer. For EGFP_1–4_, on the contrary, we observed an apparent decrease of *τ*_*D*_, to a similar extent for each oligomer. Simulation of the dependence of *τ*_*D*_ on the illumination power (Supplementary Fig. S4c,d) demonstrated that saturation and bleaching influenced *τ*_*D*_ in opposing ways. Contrary to the particle number, the effect of bleaching on the diffusion time seemed to dominate over the effect of saturation for all EGFP oligomers.

From *τ*_*D*_ we calculated the diffusion coefficient *D* according to equation (9). *D* values increased strongly with the illumination power for each oligomer (Fig. 4e). At the highest illumination power (50 μW) *D* would be overestimated by ~90%. The apparent change of *D* with illumination power results from the disparate photophysical behavior of Alexa 488 and EGFP as discussed above. To empirically eliminate the effect of photophysical processes, we linearly extrapolated the *D vs. P* graph to zero illumination power to get *D*_*FCS0*_. *D*_*FCS0*_ values corrected for temperature and viscosity differences were compared with those obtained from ultracentrifugation (*D*_*vel*_) or model prediction (*D*_*calc*_) (Table 1, Fig. 4f). As expected, *D*_*FCS0*_ decreased as the oligomer length increased. *D*_*FCS0*_ or *D*_*vel*_ vs. *n* (number of subunits) was fitted to a power function, yielding exponents of ca. −0.4 in both cases, which is larger than would be expected for spheres (−0.333), in agreement with our assumption of an elongated shape. *D*_*FCS0*_ values were ~3.6–13.5% larger than *D*_*calc*_ values, and by ~10–16% larger than *D*_*vel*_. Possible reasons for the discrepancies include uncertainties in the assumptions about the molecular shape for *D*_*calc*_, and in the fitted FCS model function.

#### Molecular brightness

**Figure 5:**
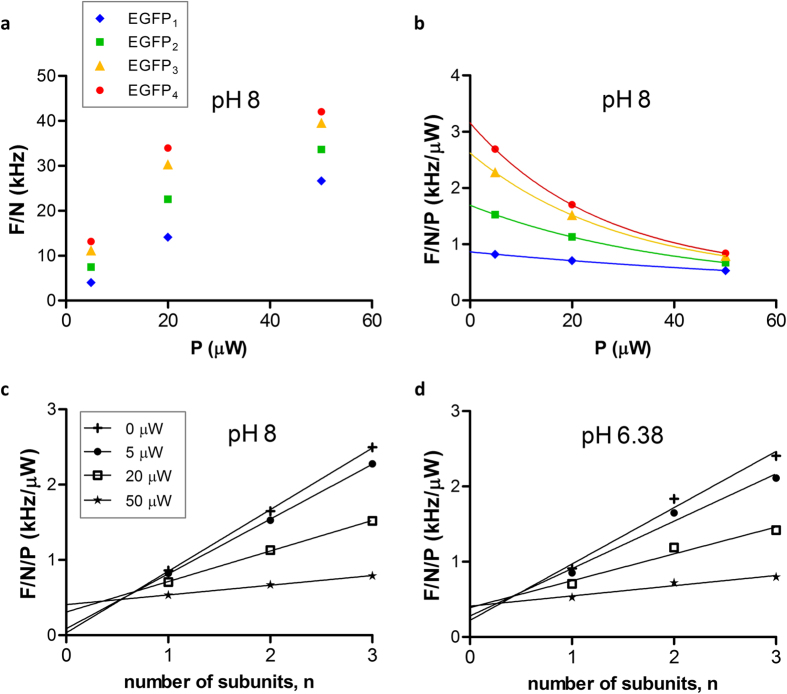
Dependence of FCS-derived molecular brightness on the oligomer size and the illumination power. (**a**) Raw molecular brightness data of EGFP_1–4_. (**b**) Molecular brightness normalized by the illumination power as a function of the illumination power. Data were fitted to exponential decays. (**c**,**d**) Molecular brightness normalized by the illumination power as a function of the number of subunits at pH 8 and 6.38. The data for zero illumination power were obtained as the amplitudes of the fits in F/N/P vs. P. From the slope and intercept of linear fits the long-lived dark fractions of EGFP were derived according to equation (5).

The molecular brightness, *F/N*, characterizes the average fluorescence intensity detected per particle, where *F* is measured directly and *N* is derived from the fit to the ACF. *F/N* can be an indicator of the association state of fluorophores or labeled molecules, but its value may be prone to photophysical artefacts. Figure 5a presents the molecular brightness as a function of illumination power for the EGFP oligomers. The increase of *F/N* was not linear especially for the higher oligomers. Correspondingly, the *F/N/P* brightness per unit illumination power decreased as a function of *P* (Fig. 5b). According to our simulations both saturation and bleaching lead to a decrease of brightness (Supplementary Fig. 4e,f). In the simulation of bleaching, the normalized brightness decreased more strongly with increasing oligomer size due to the longer dwell times. On the other hand, the effect of excitation saturation is size independent as it only depends on the fluorescence lifetime relative to the time lag between two excitation photons reaching a fluorophore. In our measurements we observed a very strong decrease of *F/N/P* at higher P values, which was larger than 3 fold for the tetramer, and less than two-fold for the monomer. This length dependence was also present in our simulations. To estimate the value of *F/N/P* free of light-induced artifacts, we made an exponential extrapolation of the graph to P = 0, which we considered as the most artifact-free brightness values, and used them in subsequent analyses.

#### Assessment of long-lived dark fraction from the apparent brightness of oligomers

In addition to light-induced processes (triplet, bleaching, light-induced blinking), fluorescent proteins can also be found in dark states due to other reasons. If a monomeric EGFP is in a dark state lasting longer than the diffusion time, it is not seen at all, leading to underestimation of the number of fluorophores or fluorescently tagged proteins. On the other hand, if one or several EGFP subunits are dark in an oligomer, but there is at least one in a bright state, the particle will still be observed, although the apparent number of particles, i.e., the 1/G(0) reciprocal amplitude of the ACF, will decrease (see Supplementary Information, equations S16–17). We calculated the effect of long-lived dark states on the measurable brightness of oligomers as shown below.

In an oligomer of fluorescent proteins (FPs), each subunit can be in a bright “on” or a long-lived dark “off” state. For the calculation of the apparent brightness we need the following parameters:

*p*: probability of an FP molecule to be in the “on” state (involving the fluorescent state, the triplet state and other short-lived dark states having an off-time shorter than τ_diff_).

1-*p*: probability of an FP molecule to be in a non-fluorescent “off” state lasting longer than the diffusion time (τ_off_>>τ_diff_).

*N*: mean number of particles in the detection volume.

*F*: mean total fluorescence.

*Ψ*: molecular brightness of a monomeric FP.

*n*: number of subunits in the FP oligomer.

*k*: number of FPs in the “on” state in a given oligomer.

In an FP oligomer composed of *n* subunits, *k* = 0, 1, …, *n* subunits can be “on”. For a mixture of different species the mean fluorescence intensity is


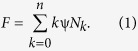


where *N*_*k*_ is the number of molecules with *k* subunits in the “on” state and *kΨ* is the molecular brightness of this species. The amplitude of the autocorrelation function is


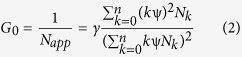


where γ is a factor depending on the geometry of the detection volume. For simplicity we set *γ* = 1. *N*_*app*_ is the reciprocal of the *G(0)* amplitude, which for samples with heterogeneous brightness is an underestimation of the total number of molecules *N*. The denominator of equation (2) is the square of the mean fluorescence intensity. The apparent molecular brightness is


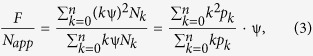


where *p*_*k*_ = *N*_*k*_/*N* is the fraction of molecules with *k* subunits in the “on” state. If the “on” or “off” state of a subunit is independent of the state of the others, we can assume that *k* follows a binomial distribution. It can then be derived (see Supplementary Information, Chapter 2) that the apparent molecular brightness is


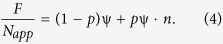


*F/N*_*app*_ is a linear function of *n* with a nonzero intercept if *p* is not unity, i.e., if there are FPs in the long-lived dark state. From the parameters of the linear fit to the *F/N*_*app*_ *vs. n* graph, we get *p* as


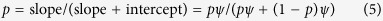


To our knowledge, this is the first time that the fraction of long-lived dark states of freely diffusing FPs are determined. The above combinatorial approach is made possible by the application of oligomers composed of different numbers of subunits.

This model was applied to fit our measurements on EGFP oligomers to assess the amount of long-lived dark species under different conditions. We were interested in the value of *p* in the absence of light-induced processes, thus we used the *F/N/P* values extrapolated for *P* = 0 μW as a measure of artefact-free brightness. In Fig. 5c *F/N/P* is shown as a function of *n* for different illumination powers. Only the results for EGFP_1–3_ are presented because the EGFP_4_ sample contained a few percent of lower oligomers (Fig. S2b), which could corrupt the linear fit. The graphs were linear for all illumination powers. The slopes decreased while the intercepts increased with increasing illumination power. The apparent probability *p* of the dark state was obtained for each illumination power the way described above. In the absence of excitation light, almost all EGFP subunits were in the “on” state, *p* = 0.96, i.e., 4% of the subunits were in long-lived dark states. In the presence of light the value of *p* decreased dramatically with the illumination power (Table 3). In this case initially bright subunits could transit to light-induced dark states (e.g. due to bleaching) during their dwell time. Thus, the assumption of the model that a given subunit is either on or off throughout its dwell time, is not fulfilled. This way only the *p* values derived from data extrapolated to zero illumination are valid indicators of the “on” state fractions.

#### Protonation induced dark state

Previous studies used EGFP monomers to investigate protonation dependent dark state formation at different pH values^8^,^13^. We used our approach based on different oligomers and extrapolation to zero light to assess the effect of a lower pH on the apparent “on” fraction. As an example, we measured the autocorrelation functions of EGFP_1–4_ at pH 6.38 with different illumination powers. Curves were fitted with equation (8) without taking the protonation process into account because a second non-fluorescent component had an insignificant fraction and did not improve the fit. The apparent probability *p* of the “on” state was calculated as detailed above for pH 8. The value of *p* was 0.77 for zero light extrapolation. This corresponds to a dark state fraction of 23%, which includes the long-lived dark states and the additional protonation-induced dark states. Haupts *et al*.^8^ also reported ~3–4% and ~20–25% protonated EGFP fraction from excitation spectra at pH 8 and pH 6.4. Widengren at al^13^ measured ~35% protonation induced dark state fraction by FCS at pH 6 at buffer conditions similar to ours. The characteristic time constants of protonation-deprotonation processes are in the hundred μs to ms range^8^,^13^, which are close to the dwell times (100–200 μs). Hence, transition between the protonated and deprotonated states could take place during the dwell time (such as for the case of bleaching) thereby affecting the ACF amplitude and the apparent brightness. Thus, our model assumptions are not fulfilled, and the derived *p* values are not exact. Apparent values of *p* decreased with increasing illumination power probably due to bleaching and saturation such as for pH 8. To determine the fraction of long-lived dark states, measurements at several low illumination powers and extrapolation to zero light are recommended.

## Discussion

Oligomers of fluorescent proteins were first introduced to increase the brightness of tagged proteins in live cells^25^,^26^,^27^. Our group mapped the intracellular mobility of EGFP_1–4_ oligomers by single-point FCS^5^ and to study accessibility of nuclear regions. Different fluorescence methods (FCS, Number and Brightness Analysis or FRET) allowed the identification of oligomerization of various FP-tagged proteins (e.g. in refs 3,6,28,29). We propose that investigation of the fluorescence properties of pure EGFP oligomeric standards would help to interpret results obtained with oligomerized EGFP fusion proteins of biological interest.

The hydrodynamic and photophysical properties of EGFP have been studied by several labs. The corrected sedimentation coefficient of the EGFP monomer derived from AU-FDS was 2.68 S in ref. 30, and 2.52 S for our measurement, whereas the calculated value was 2.6 S. Macgregor *et al*.^16^ compared results from analytical ultracentrifugation, AUC, (2.81 S) and AU-FDS (2.73 S) on His tagged EGFP. Petrasek *et al*.^31^ used scanning FCS and received 92.3 μm^2^/s (corrected for 20°), and Widengren *et al*. measured 85 ± 4 μm^2^/s by FCS at room temperature^12^. FRAP yielded 87 ± 16 μm^2^/s (at 20 °C)^32^. In this study, we applied AU-FDS, a frequently used method for diffusion coefficient determination, which resulted in D_(w,20 °C)_ = 83.4 ± 0.5 μm^2^/s, and FCS, which yielded *D*_(w,20 °C)_ = 97.3 ± 5 μm^2^/s after extrapolation to zero excitation light. It seems that FCS-determined *D* values are systematically higher than those obtained from AUC. Fluorescence of EGFP monomers is affected by several light and protonation induced processes. For triplet correlation times we obtained values in the microsecond range, which decreased with increasing illumination power. The increase of illumination power resulted in the increase of the apparent diffusion coefficient as a net effect of photobleaching (increasing the apparent *D*) and excitation saturation (reducing the apparent *D*)^11^,^12^. The dominance between these two processes is dye dependent: for fluorescein, Rh6G^33^, similarly to EGFP, bleaching seems to dominate, contrary to the more photostable Alexa 488 (Fig. 4d). Due to this disparate behavior, measuring the Alexa 488 standard at the same laser power does not compensate the error of *D* due to bleaching; on the contrary, it strengthens this effect.

The hydrodynamic and fluorescence properties of purified EGFP oligomers have been systematically investigated in this study. The obtained sedimentation coefficients allowed us to identify the most probable, “barrels standing in a row”, arrangement of the subunits. Whereas the decrease of diffusion coefficients with EGFP oligomerization degree was similar in AU-FDS and FCS detection, FCS-derived *D* values were systematically higher for each oligomer. FCS-derived D values taken from earlier studies^5^,^14^ are within 5–20% of ours after correction for differences in temperature, and show a similar dependency on oligomer length.

The dependence of fluorescence properties of the oligomers on the illumination power can only partially be explained by the properties of the individual subunits. The measured apparent *N* values (representing the concentration) did not vary significantly for the monomers, and increased with increasing P for oligomers, to an extent proportional to the oligomer size. Excitation saturation and bleaching of the subunits was not sufficient to explain this size dependent behavior. The normalized molecular brightness (F/N/P) decreased more strongly for larger oligomers, in accordance with our simulations. Apparent D values increased substantially for monomers with illumination power, and less strongly for oligomers (in agreement with simulations) because if not all subunits are bleached, the oligomer is still detectable.

By now we considered only light-induced dark states of the subunits. Fluorescent proteins can also be in dark states due to other reasons such as imperfect dye maturation or protonation. Since the individual subunits in an oligomer may be in “on” (bright) or “off” (dark) states, the FCS-determined apparent brightness (F/N) is not an integer multiple of that of the monomer. From the length dependence of F/N we determined the p “on” state probability of the subunits. The apparent value of p decreased with illumination power. To eliminate the effect of light induced dark states, we extrapolated to zero excitation light, and found that only 4% of the subunits were in long-lived dark states at pH 8. Madl *et al*. reported 12% dark state (not corrected for the effect of illumination) for GFP tagging CRAC channels expressed in the cell membrane^28^.

pH-dependence of excitation spectra was used earlier to calculate the protonated nonfluorescent fraction of EGFP monomers, e.g. in ref. 8. Widengren *et al*.^12^ introduced a model function to account for protonation induced blinking in FCS. Our results on the long lasting dark fractions are in accurate agreement with their data. Since our method detects long lasting dark states, the subunits in the purified recombinant oligomers probably do not reside in dark states other than the protonated state. However, in live cells the expression of FP-labeled proteins is continuous, and immature, nonfluorescent species are also present and participate in protein complexes, which could not be detected by using the earlier methods. Our proposed procedure can assess the fraction of “on” and “off” states (by expressing FP oligomers in cells under similar conditions as the FP-tagged proteins of interest), and make the quantitation of oligomerization more accurate.

## Materials and Methods

### Preparation and purification of EGFP_1–4_ oligomers

EGFP_1–4_ are multimeric fluorescent proteins, with 5-amino-acid (G-P-V-A-T) linkers connecting the EGFP units. The plasmids encoding for EGFP_1–4_ were a kind gift of Dr. M. M. Nalaskowski (Universitätsklinikum Hamburg-Eppendorf)^25^,^26^. For stable transfection, multimeric EGFP inserts, EGFP_n_^5^ were cloned into a pSV vector originating from promoterless pECFP-1 (Clontech). SV40 promoter sequence was inserted at the Hind III restriction site to drive overexpression. HeLa cells were cultivated in DMEM without phenol red, containing 10% fetal calf serum and 200 mM L-glutamine in humidified 5% CO_2_ atmosphere at 37 °C. The cells were transfected with the pSV-EGFP_n_ vector according to the Fugene HD user manual (Roche, Germany). Stably transfected cells were treated with G418 (Geneticin, Invitrogen, Life Technology, CA, USA) at a final concentration of 50 μg/ml and clones with strong EGFP expression were selected. For protein purification cells were seeded in petri dishes 72 h before harvesting. Cells were collected, homogenized (“dounced”), treated with 0.2% Nonidet P40 (Shell Chemicals) and centrifuged at 1200 × g for 10 min. The white pellet was removed and discarded, the green supernatant was incubated with DNase (5 μg/ml) and RNase (30 μg/ml) for 15 min. EGFP_1–4_ proteins were purified on 1 ml DEAE Sepharose Fast Flow columns (GE Healthcare). The column was washed with 10 ml 20 mM Tris pH 8, 50 mM NaCl. Proteins were eluted in 1 ml 20 mM Tris pH 8, 150 mM NaCl. In the SDS gel the oligomers appear as unique bands according to their expected masses (Fig. S2A). Homogeneity of the oligomers was checked by fluorescence detection in native polyacrylamide gel electrophoresis (Fig. S2B). Proteins were stored in this buffer with protease inhibitor (complete mini, EDTA free, Roche) and 0.01% Nonidet P40 at −80 °C. In all experiments 20 mM Tris-HCl, 100 mM NaCl, pH 8 buffer was used, unless specified otherwise.

### Absorption and fluorescence characteristics

Absorption spectra of the purified EGFP_1–4_ samples were measured on Varian Cary-4E spectrophotometers. The fluorophore concentration was calculated using an extinction coefficient of 56,000 M^−1^cm^−1^ at 489 nm supposing additivity^34^.

Fluorescence spectra were recorded using an SLM 810 spectrofluorimeter equipped with a 450 W Xenon lamp. Samples were excited at 470 nm and spectra recorded at a bandwidth of 4 nm in the 480–650 nm wavelength range. The presented spectra were corrected for the instrument characteristics. Anisotropy was measured in the same setup using Glan-Thompson prisms to polarize and analyze the light. Fluorescence lifetime measurements were performed on a FluoTime 100 (Picoquant, Germany) instrument.

### Structural models of the EGFP_1–4_

The molar masses *M*_*calc*_ and partial specific volume (*V*_*spec*_ = 0.732 l/kg of EGFP_1–4_ according to the amino acid sequence) were calculated with the program SEDNTERP ver. 1.09^35^. The three-dimensional structure of the EGFP monomer was taken from the Protein Data Bank^20^. The 5 amino-acid-linkers between the monomers were taken into account in the calculation. Different arrangements of the oligomers were built with the help of the program *Wincoot*^36^. Sedimentation coefficients *s*_*calc*_ and diffusion coefficients *D*_*calc*_ for the cylinder-shaped EGFP and its different oligomeric arrangements were calculated with the program HYDROPRO ver. 5a^37^.

### Analytical ultracentrifugation with fluorescence detection system (AU-FDS)

AU-FDS runs were carried out in a ProteomeLab XL-I analytical ultracentrifuge (Beckman Coulter, Indianapolis, IN) equipped with a fluorescence detection system (AU-FDS; Aviv Biomedical Inc., Lakewood, NJ), using An-50 Ti and An-60 Ti rotors. Excitation was at 488 nm using a 10 mW solid-state laser; emission was detected through a dichroic mirror and a 505–565 nm bandpass filter. The centrifuge was programmed and data were recorded using AOS software (Aviv Biomedical Inc., Lakewood, NJ). Special cell housings (Nanolytics, Potsdam, Germany) allowed the placement of 3 mm double sector centerpieces directly beneath the upper window of the cell. All runs were done in 20 mM Tris/HCl pH 8.0, 150 mM NaCl, 1 mM DTT, 0.01% NP40.

Sedimentation velocity runs were done in charcoal-filled epon double-sector centerpieces at 50,000 rpm and 20 °C using a sample volume of 100 μl. Scans were recorded with a radial increment of 20 μm and were averaged over 5 rotor revolutions. Data were analyzed with the program DCDT + (ver. 2.3.2)^19^,^38^, which implements the algorithms described in ref.^18^,^39^ to obtain the sedimentation and diffusion coefficients (*s*_*vel*_ and *D*_*vel*_). All values were corrected to water as solvent. The molar masses (*M*_*vel*_) of the EGFP oligomers were calculated through the Svedberg equation as:


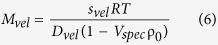


where *R* is the gas constant, *T* is the absolute temperature, *ρ*_*0*_ is the density of the buffer and *V*_*spec*_ is the partial specific volume of EGFP_1–4_.

Sedimentation equilibrium runs were done at 4 °C in titanium double-sector centerpieces filled with 40 μl sample at 7000, 11000 and 18000 rpm and three different concentrations of the proteins (20, 50 and 150 nM). Data were measured with a radial increment of 5 μm and were averaged over 10 rotor revolutions. After no further change in the concentration distribution could be observed, scans at equilibrium were averaged and molar masses were determined by globally fitting the data at all rotor speeds with a single species model using the program package BPCFIT^15^:


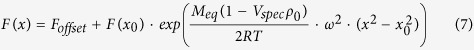


Here *x* is the distance from the center of rotation, *F(x)* and *F(x*_*0*_) are the fluorescence intensities at positions *x* and *x*_*0*_, *ω* the angular velocity of the rotor and *M*_*eq*_ the molar mass. Baseline offset (*F*_*offset*_) was set to the buffer fluorescence measured in each cell near the meniscus after sedimenting the proteins for 16 h at 44000 rpm at the end of the experiment.

### Fluorescence Correlation Spectroscopy

To measure diffusion properties of EGFP oligomers, we used fluorescence correlation spectroscopy (FCS). The FCS microscope is described in ref. 6. The instrument is based on an Olympus FluoView 1000 confocal microscope (UPlanAPO 60× water immersion objective, NA 1.2) equipped with an FCS extension (Steinbeis Center for Biophysical Analysis, Heidelberg, Germany). For excitation, the 488 nm line of an Ar ion laser was used at different intensities (5, 20, 50 μW at the objective corresponding to ~40–400 MW/m^2^); fluorescence emission was detected through a 500–550 nm band-pass filter by an avalanche photodiode (Perkin-Elmer). The autocorrelation function of the signal was calculated by an ALV-5000E correlator (ALV-Laser GmbH, Langen, Germany). Runs of 5 × 20 seconds were recorded. Autocorrelation curves were fitted by using the software Quickfit 3.0^40^ to a model assuming 3D diffusion of a single component plus triplet state formation:


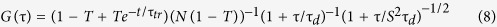


where τ is the lag time, *T* is the equilibrium fraction of dyes in the triplet state, *τ*_*tr*_ is the triplet correlation time, τ_*d*_ is the diffusion time (the average dwell time of the molecule in the detection volume), *N* is the average number of molecules in the detection volume and *S* is the axial ratio of the ellipsoidal detection volume. The diffusion coefficient was calculated using the following expression:


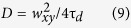


where *w*_*xy*_ is the lateral radius of the detection volume (where detection efficiency is e^−2^ times that of the maximum); *w*_*xy*_ was calibrated (using equation (9)) with a solution of Alexa-488 having a known diffusion coefficient (435 μm^2^/s at 22.5 °C)^31^, yielding *w*_*xy*_~208 nm when extrapolating its diffusion time to zero laser power.

## Additional Information

**How to cite this article**: Vámosi, G. *et al*. EGFP oligomers as natural fluorescence and hydrodynamic standards. *Sci. Rep.* **6**, 33022; doi: 10.1038/srep33022 (2016).

## Supplementary Material

Supplementary Information

## Figures and Tables

**Table 1:** Comparison of molar mass and hydrodynamic parameters obtained from different techniques.

*n*	*M*_*calc*,_ (kg/mol)	*s*_*calc*_(S)	*D*_*calc*_ (μm^2^/s)	*M*_*eq*_ (kg/mol)	*s*_*vel*_ (S)	*D*_*vel*_ (μm^2^/s)	*M*_*vel*_ (kg/mol)	*D*_*FCS0*_ (μm^2^/s)
1	26.9	2.6	88.8	26.7 ± 0.2	2.52 ± 0.02	83.4 ± 0.5	27.5 ± 0.3	97.3 ± 5
2	54.3	3.9	67.4	51.5 ± 0.15	3.73 ± 0.03	63.7 ± 0.9	53.4 ± 1	69.8 ± 4
3	81.7	5.0	56.9	77.8 ± 0.1	4.75 ± 0.05	54.1 ± 2	80 ± 3	60.2 ± 3
4	109.1	5.65^rod^	48.3	98.4 ± 0.45	5.54 ± 0.06	48.2 ± 0.7	105 ± 2	54.8 ± 3
		6.02^star^						
		6.15^square^						

Errors denote s. d. All data were corrected to 20 °C and water as solvent. *n* refers to the number of EGFP subunits in the oligomers.

*M*_*calc*_ values were obtained from the sequence, *s*_*calc*_ and *D*_*calc*_ were calculated according to Materials and methods. Rod, star and square indexes refer to the arrangement of the subunits (see Fig. 2).

*M*_*eq*_ was obtained from sedimentation equilibrium, *s*_*vel*_ and *D*_*vel*_ are mean values from sedimentation velocity runs.

*M*_*vel*_ was calculated from *s*_*vel*_ and *D*_*vel*_.

*D*_*FCS0*_ was obtained from fits to FCS measurements, extrapolated to zero laser intensity.

**Table 2:** Bulk fluorescence properties of EGFP oligomers.

	Fluorescence lifetime (ns)	Fluorescence anisotropy
EGFP_1_	2.885 ± 0.016	0.32 ± 0.01
EGFP_2_	2.776 ± 0.013	0.27 ± 0.01
EGFP_3_	2.755 ± 0.013	0.24 ± 0.01
EGFP_4_	2.744 ± 0.013	0.23 ± 0.01

**Table 3:** Probability of “on” (bright) state of EGFP subunits at two pH values and different illumination powers.

Laser intensity (μW)	p(on)	
	pH 8	pH 6.38
0*	**0.96**	**0.77**
*5*	*0.89*	*0.69*
*20*	*0.57*	*0.48*
*50*	*0.24*	*0.25*

^*^Refers to extrapolation to zero light conditions.
