# Supplementary material for: EGFP oligomers as natural fluorescence and hydrodynamic standards
Source: Sci Rep. 2016 Sep 13;6:33022. doi: 10.1038/srep33022 (PMC5020695; doi:10.1038/srep33022)
Supplement: Supplementary Information [file srep33022-s1.pdf]

SUPPLEMENTARY INFORMATION

EGFP oligomers as natural fluorescence and hydrodynamic standards

György Vámosi, Norbert Mücke, Gabriele Müller, Jan Wolfgang Krieger, Ute Curth, Jörg Langowski, Katalin Tóth

Chapter 1.
Simulation of photophysical effects on the autocorrelation curves

Simulations were performed with a home-built FCS simulation software, available as open-source software from https://github.com/jkriege2/FCSSimulator. In brief, this software simulates a simple 3D random walk for several particles in a spherical box with a diameter of 8 µm. The single-direction jump length Δx in each time step Δt_{sim}=1 µs is distributed normally around Δx=0 with an s.d. of $\sqrt{2D\Delta t_{sim}}$, where D is the diffusion coefficient fixed to 90, 70, 60, 50 µm²/s for $EGFP_1$, $EGFP_2$, $EGFP_3$ and $EGFP_4$. These values are approximately equal to D_{calc} given in Table 1 in the main text. The particle concentration was set to 80 nM. To keep this concentration constant over time, a particle is deleted, when it leaves the simulation box, and a new particle is introduced at a random position at the surface of the box. Depending on the simulated molecule, 1-4 independent fluorophores are assigned to each random walker. Each of these fluorophores can bleach irreversibly and independently with a probability $p_{bleach} = \Delta t_{sim} / \tau_{bleach}$, where τ_{bleach} is the 1/e bleach-lifetime at the center of the illumination focus, where the laser intensity is maximal. τ_{bleach} is inversely proportional to the laser intensity, so it remains unbleached longer at the borders of the illumination focus. If a bleached (or unbleached) fluorophore leaves the simulation box, the newly created particle that replaces it is in a non-bleached state again. This keeps the average particle number in the simulation box constant, and models an infinite reservoir of intact fluorophores outside the confocal observation volume. This is a good model for a small laser focus (femtoliter volume) in a reservoir (a few hundred microliters of sample in a sample chamber), which is several orders of magnitude larger. Finally, in each step, the detection of fluorescence from all particles in the simulation box is simulated. To this end, a Gaussian detection volume

$$\mathrm{MDE}(x,y,z) = \exp\left(-2\frac{x^2+y^2}{w_{\mathrm{xy}}^2} - 2\frac{z^2}{w_{\mathrm{z}}^2}\right), \qquad \text{(S1)}$$

with w_{xy}=300 nm lateral and w_{z} = 1200 nm longitudinal 1/e² radii is used. Then the expected number of fluorescence photons from this focal volume is calculated as a sum over all visible (non-bleached) fluorophores $i = 1\ldots N_{visible}$ at their current positions $x_i(t)$, $y_i(t)$, $z_i(t)$:

$$\overline{N_{\mathrm{Photons}}}(t) = \sum_{i=1}^{N_{\mathrm{visible}}} N_{\mathrm{abs}} q_{\mathrm{fluor}} q_{\mathrm{det}} \underbrace{\left[\mathrm{MDE}\left(x_i(t), y_i(t), z_i(t)\right)\right]^2}_{illumination+detection} \qquad \text{(S2)}$$

Here N_{abs}~6.4 is the number of photons that is absorbed on average by a single fluorophore during Δt_{sim}=1 µs, the fluorescence quantum yield is q_{fluor} = 0.92 and the detection efficiency is q_{det} = 1. The quantity $\bar{N}_{Photons}(t)$ is accumulated over two subsequent time steps Δt_{sim}, and then the number of detected photons $N_{phot,det}(t)$ is drawn as a random number from a Poissonian distribution with the accumulated $\bar{N}_{Photons}(t)$ as mean. From this random sequence of detected photons the simulation software then calculates the FCS correlation function:

$$g(\tau) = \frac{\left\langle N_{phot,det}(t) \cdot N_{phot,det}(t+\tau) \right\rangle}{\left\langle N_{phot,det}(t) \right\rangle^2}, \tag{S3}$$

which is then evaluated just as the experimental curves using the software QuickFit 3.0 [1].

Simulation of intensity-dependent bleaching: According to the above model the bleach-process does not depend on the laser intensity at the position $x_i(t)$, $y_i(t)$, $z_i(t)$ of a fluorophore *i*. Because bleaching occurs in the excited state of the fluorophore, the above model needs to be modified to account for the number of photons absorbed by a fluorophore at its current position:

$$N_{abs,i}(t) = N_{abs} \cdot MDE\left(x_i(t), y_i(t), z_i(t)\right). \tag{S4}$$

Then the probability of bleaching, which was initially determined by the average lifetime τ_{bleach} of the on-state of a fluorophore, is modified as follows:

$$p_{bleach} = \frac{\Delta t_{sim}}{\tau_{bleach}} \xrightarrow[\text{modified}]{} p_{bleach} = \frac{\Delta t_{sim}}{\tau_{bleach}} \cdot f_{bleach} \cdot MDE\left(x_i(t), y_i(t), z_i(t)\right) \tag{S5}$$

where f_{bleach} is a dimensionless scaling factor, which was set to 10 for all simulations. This factor simply adapts the range of values of the MDE ($MDE(x, y, z) \in [0,1]$) to a reasonable probability for the simulation, as compared to the case of position-independent bleaching. The same effect could have been reached by altering the lifetime τ_{bleach} itself. As we do not interpret the absolute value of τ_{bleach} quantitatively, only its relative changes, this does not influence our interpretation of the data.

Simulation of the effect of excitation saturation: In order to simulate the saturation of the fluorescence excitation transition, the sum over all visible fluorophores is modified as follows with a relative saturation intensity α_{sat} [2]:

$$\bar{N}_{Photons}(t) = \sum_{i=1}^{N_{visible}} N_0 \cdot q_{fluor} \cdot q_{det} \cdot \underbrace{\frac{\alpha_{sat} \cdot MDE\left(x_i(t), y_i(t), z_i(t)\right)}{\alpha_{sat} + MDE\left(x_i(t), y_i(t), z_i(t)\right)}}_{\text{saturated excitation}} \cdot \underbrace{MDE\left(x_i(t), y_i(t), z_i(t)\right)}_{\text{detection}} \tag{S6}$$

For real fluorophores, the saturation is usually described by the fluorophore's saturation intensity I_{sat}. For the simulations, described herein, the absolute saturation intensity is not important and it is replaced by the dimensionless saturation parameter $\alpha_{sat} = I_{sat}/I_0$, which determines I_{sat} relative to the maximum illumination intensity I_0 = 22 kW/cm^2 in the center of the MDE (corresponding to a laser power of 250 µW at the objective).

Note that all simulations shown in this paper were performed in order to separate the effects of bleaching and saturated excitation, i.e., we always simulated only one of the two effects exclusively. Therefore, there is no saturation-effect in the bleaching process and vice versa. Including such an effect would require the calibration of both the saturation intensity and the bleaching lifetime to real experimental values, which is out of the scope of this paper. Accordingly, we did not interpret the absolute values of the parameters τ_{bleach} and $\alpha_{sat} = I_{sat}/I_0$, and did not relate them to each other quantitatively. However, the parameterization of the simulations was chosen in such a way that the relative changes in τ_{bleach} and $\alpha_{sat} = I_{sat}/I_0$ reflect the behavior expected from real experiments, and therefore allow for a comparison with them.

Chapter 2.
Generalized calculation of the apparent molecular brightness of FP oligomers having long-lived dark states

For a mixture of *n* different species in an FCS experiment, the mean fluorescence intensity is

$$F = \sum_{k=1}^{n} \psi_k N_k \ . \qquad \text{(S7)}$$

where N_k is the number of molecules and Ψ_k is the molecular brightness of the *k*-th species. The amplitude of the correlation function is

$$G(0) = \frac{1}{N_{app}} = \gamma \frac{\sum_{k=1}^{n} \psi_k^2 N_k}{\left(\sum_{k=1}^{n} \psi_k N_k \right)^2} \qquad \text{(S8)}$$

where N_{app} is the apparent number of molecules in the detection volume, and γ is a factor depending on the geometry of the detection volume. For the sake of simplicity we set $\gamma=1$. The denominator of equation (S8) is the square of the mean fluorescence intensity. The apparent molecular brightness is

$$\frac{F}{N_{app}} = \frac{\sum_{k=1}^{n} \psi_k^2 N_k}{\sum_{k=1}^{n} \psi_k N_k} = \frac{\sum_{k=1}^{n} \psi_k^2 p_k}{\sum_{k=1}^{n} \psi_k p_k} \qquad \text{(S9)}$$

where p_k is the fraction of molecules in the *k*-th state.

For the calculation of the apparent brightness of an FP oligomer, we need the following parameters:

- 1-*p*: probability of an FP molecule to be in a non-fluorescent “off” state lasting longer than the diffusion time ($\tau_{off} >> \tau_{diff}$)
- *p*: probability of an FP molecule to be in the “on” state (involving the fluorescent state, the triplet state and other short-lived dark states having an off-time shorter than τ_{diff})
- *N*: mean number of particles in the detection volume
- *F*: mean total fluorescence
- Ψ: molecular brightness of a single FP (F/N)
- *n*: number of subunits in the FP oligomer
- *k*: number of FPs in the on state in a given oligomer

In an FP *n*-mer 0, 1, …,*n* FP molecules can fluoresce. The probability that in an *n*-mer exactly *k* FPs are in the “on” state is given by a binomial formula:

$$p_k = \binom{n}{k} p^k (1-p)^{n-k} \qquad \text{(S10)}$$

The brightness of a species with *k* FPs being in the “on” state is $\Psi_k = k\Psi$. Example for n=3:

Species	Brightness Ψ_k	Fraction p_k
○○○	3Ψ	p^3
○○●	2Ψ	$3p^2(1-p)$
○●●	1Ψ	$3p(1-p)^2$
●●●	0	$(1-p)^3$

Equation (S9) takes the following form:

$$\frac{F}{N_{app}}=\frac{\sum_{k=0}^{n}\psi_k^2 p_k}{\sum_{k=0}^{n}\psi_k p_k}=\frac{\sum_{k=0}^{n}k^2\psi^2 p_k}{\sum_{k=0}^{n}k\psi p_k}=\frac{\sum_{k=0}^{n}k^2 p_k}{\sum_{k=0}^{n}kp_k}\cdot\psi \tag{S11}$$

The sum $\sum_{k=0}^{n}kp_k$ in the denominator is the expectation value *E(X)* of a binomially distributed variable *X*, while the sum $\sum_{k=0}^{n}k^2 p_k$ in the numerator is the expectation value $E(X^2)$ of the variable X^2. The expectation value of a binomial distribution is

$$\mathrm{E}(X)=np\,. \tag{S12}$$

Using the relation

$$\sigma^2(X)=\mathrm{E}(X^2)-\mathrm{E}^2(X) \tag{S13}$$

and considering that the variance of the binomial variable *X* is

$$\sigma^2=np(1-p), \tag{S14}$$

we get the following expression for the apparent brightness:

$$\frac{F}{N_{app}}=\frac{\mathrm{E}(X^2)}{\mathrm{E}(X)}\psi=\frac{\mathrm{E}^2(X)+\sigma^2(X)}{\mathrm{E}(X)}\cdot\psi=\frac{(np)^2+np(1-p)}{np}\cdot\psi=\left[1+(n-1)p\right]\cdot\psi \tag{S15}$$

The effects of fast photophysical transitions such as triplet formation, which have shorter off-times (2-30 µs) than the diffusion time, average out during the diffusion time, therefore they do not affect the relation between the apparent mean brightness values of the different oligomers.

Calculation of the real number of particles N_{real} from N_{app} and *p*:

Equation (S8) can also be written in the form:

$$G(0)=\frac{1}{N_{app}}=\frac{\sum_{k=1}^{n}\psi_k^2 N_k}{\left(\sum_{k=1}^{n}\psi_k N_k\right)^2}=\frac{\sum_{k=1}^{n}k^2 p_k N_{real}}{\left(\sum_{k=1}^{n}kp_k N_{real}\right)^2}=\frac{1}{N_{real}}\cdot\frac{\sum_{k=1}^{n}k^2 p_k}{\left(\sum_{k=1}^{n}kp_k\right)^2}=\frac{1}{N_{real}}\cdot\frac{\mathrm{E}(X^2)}{\mathrm{E}^2(X)}$$
$$=\frac{1}{N_{real}}\cdot\frac{\mathrm{E}^2(X)+\sigma^2(X)}{\mathrm{E}^2(X)}=\frac{1}{N_{real}}\cdot\frac{(np)^2+np(1-p)}{(np)^2}=\frac{1}{N_{real}}\cdot\left(1+\frac{1-p}{np}\right) \tag{S16}$$

The total number N_{real} of FP oligomers in the detection volume is:

$$N_{real} = \frac{1}{G(0)} \cdot \left(1 + \frac{1-p}{np}\right). \qquad \text{(S17)}$$

Supplementary Citations

1 Krieger J. W., Langowski J.: QuickFit 3.0 (status: beta, compiled: Jan 5, 2015, SVN: 3695): A data evaluation application for biophysics, http://www.dkfz.de/Macromol/quickfit/ v. 3.0 (status: beta, compiled: Oct 2015) (2015).

2 Visscher, K., Brakenhoff, G. J. & Visser, T. D. Fluorescence Saturation in Confocal Microscopy. *J Microsc-Oxford* **175**, 162-165 (1994).

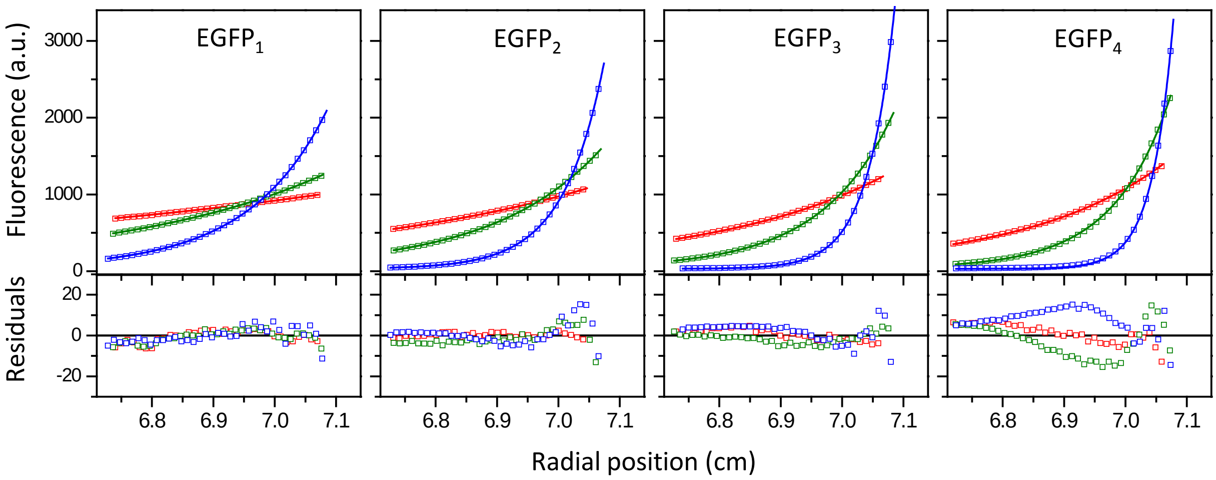

Supplementary Figure S1

Sedimentation equilibrium centrifugation using fluorescence detection.

The top panels show the radial concentration distributions. Centrifugation was carried out at 7000 (red), 11000 (green) and 18000 rpm (blue). The concentration of $EGFP_1$ was 150 nM, the concentrations of the other samples were set to yield the same fluorescence intensity. For each EGFP oligomer data recorded at different rotor speeds were globally fitted to single exponentials with fixed baselines (solid lines). The bottom panels present the residuals. M_{eq} values were derived as described.

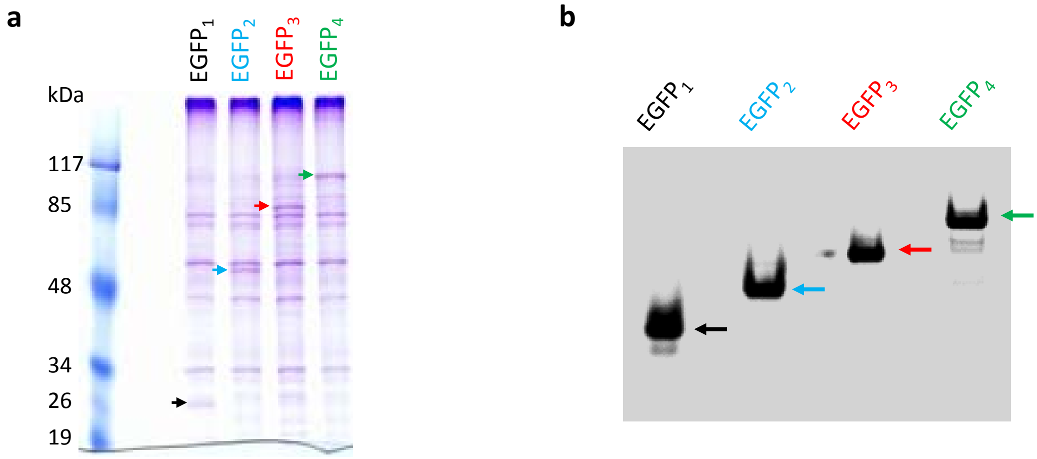

Supplementary Figure S2

Gel electrophoresis of isolated EGFP oligomers

a) Denaturing 10% SDS gel of EGFP oligomers. HeLa cells expressing the oligomers were dounced, treated with DNAse/RNAse, centrifuged, and the supernatant was purified with DEAE Sepharose Fast Flow columns.

b) Native polyacrylamide gel of $EGFP_{1\text{-}4}$ visualized with a Typhoon scanner. Proteins were assayed on a 10% native polyacrylamide gel with 0.01% SDS and 0.01% Nonidet P40. The gel was run in 320 mM glycine, 0.01% SDS, 0.01% Nonidet P40, 25 mM Tris, pH 8.8. The in–gel fluorescence of the $EGFP_{1\text{-}4}$ samples was visualized on a Typhoon-Scanner 9410 (GE Healthcare) using a 488 nm laser for excitation and 520 ± 20 nm filter for detection.

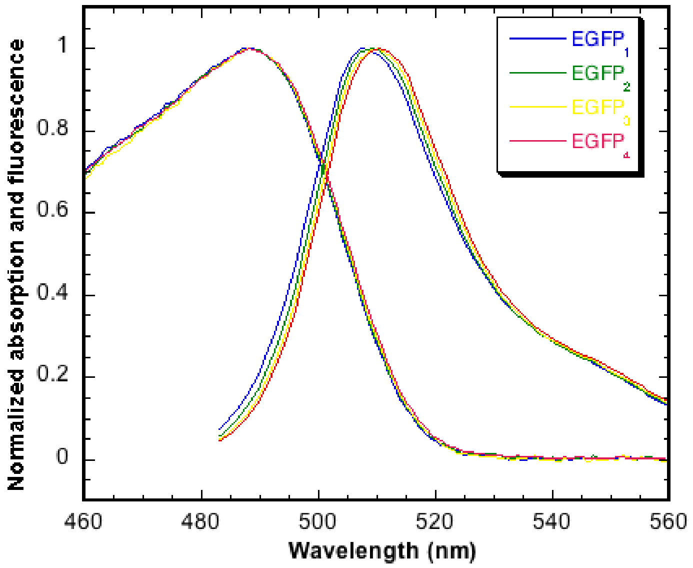

Supplementary Figure S3

Absorption and fluorescence emission spectra of $EGFP_{1\text{-}4}$ oligomers.

Samples were excited at 470 nm, and spectra were corrected for instrument characteristics. The red shift in the emission maxima from the monomer to the tetramer was 2 nm.

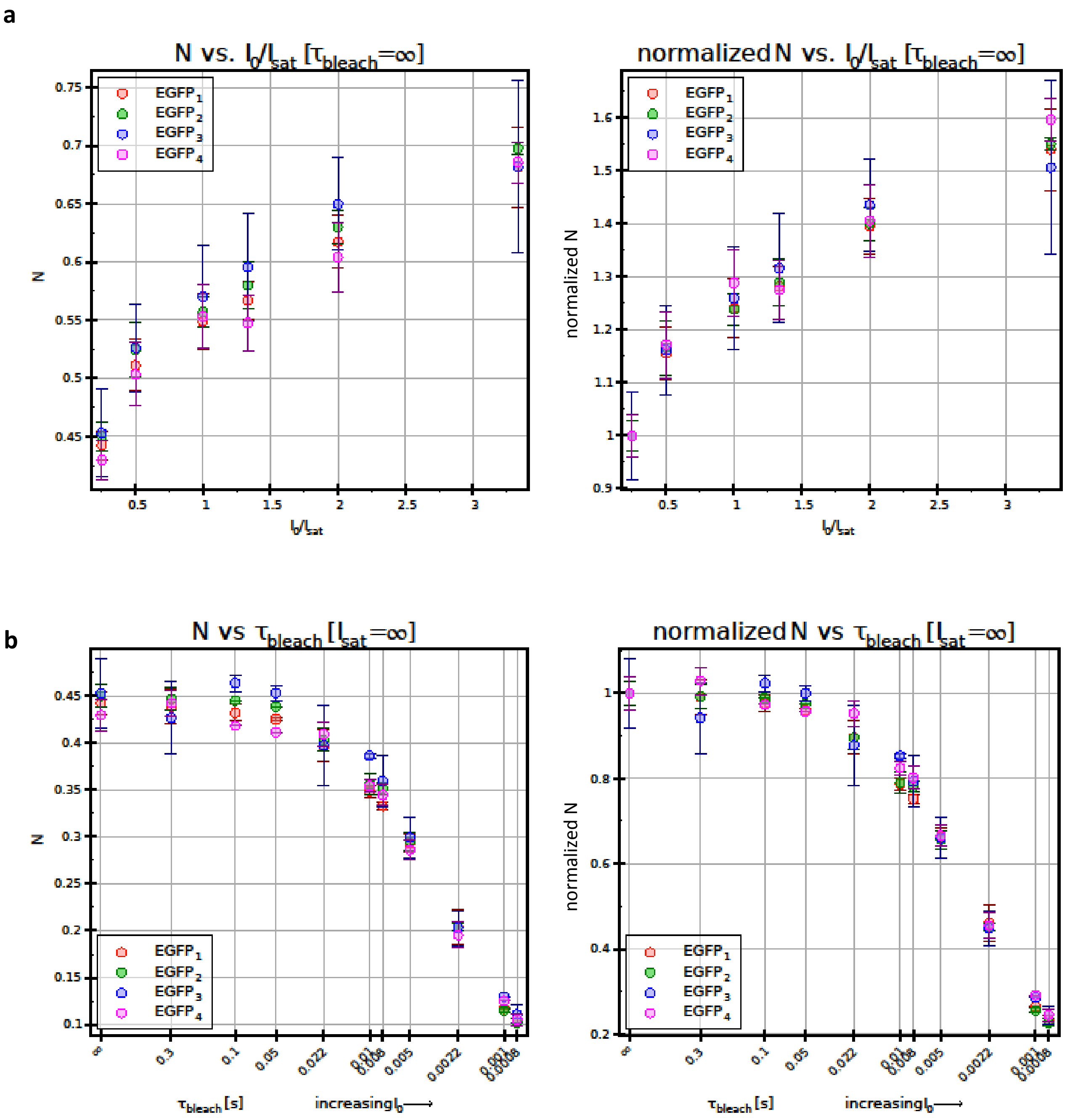

Supplementary Figure S4

Simulation of the effect of photobleaching and excitation saturation on the parameters of $EGFP_{1\text{-}4}$ oligomers.

Autocorrelation function were simulated according supplementary equation (S3), and parameters were determined by nonlinear fitting of the simulated curves to equation (8) in the main text. **a)** Apparent particle number N (left) and its normalized value (right) vs. the illumination power I_0 relative to the saturating power I_{sat} in the absence of bleaching. **b)** Apparent N vs. time-constant of bleaching (τ_{bleach} is the 1/e bleach-lifetime at the center of the illumination focus, where the laser intensity is maximal)

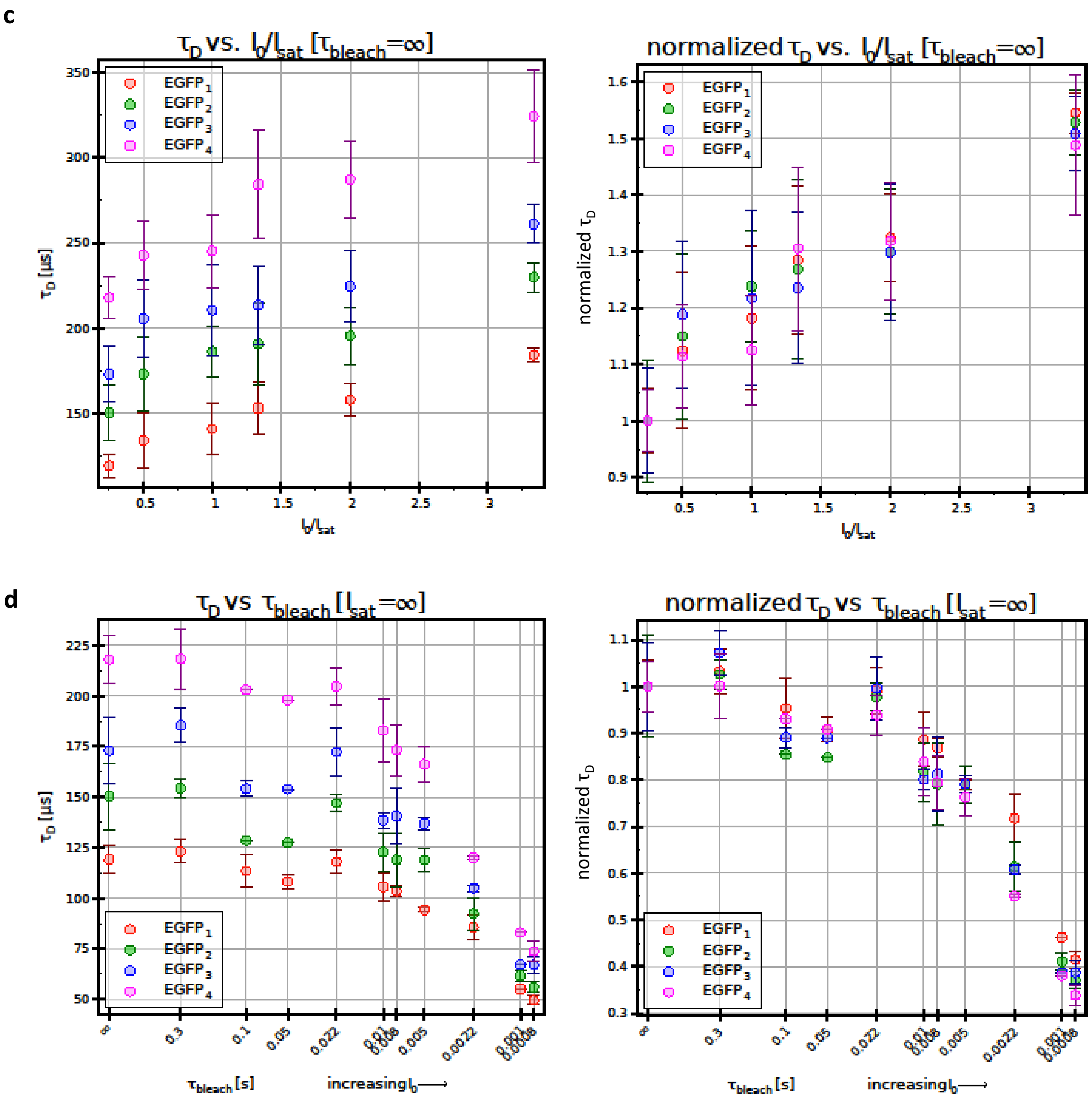

Supplementary Figure S4 (continued)
Simulation of the effect of photobleaching and excitation saturation on the parameters of $EGFP_{1-4}$ oligomers.
c) Apparent diffusion time (left) and its normalized value (right) vs. the illumination power I_0 relative to the saturating power I_{sat} in the absence of bleaching. **d)** Apparent diffusion time vs. τ_{bleach}.

e

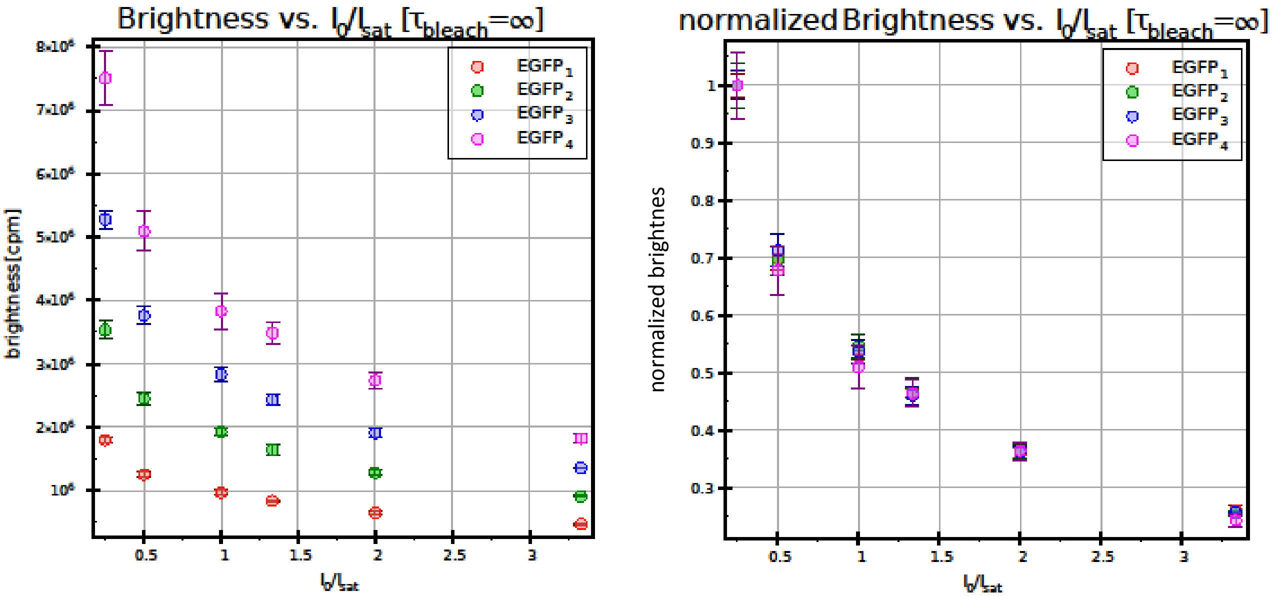

f

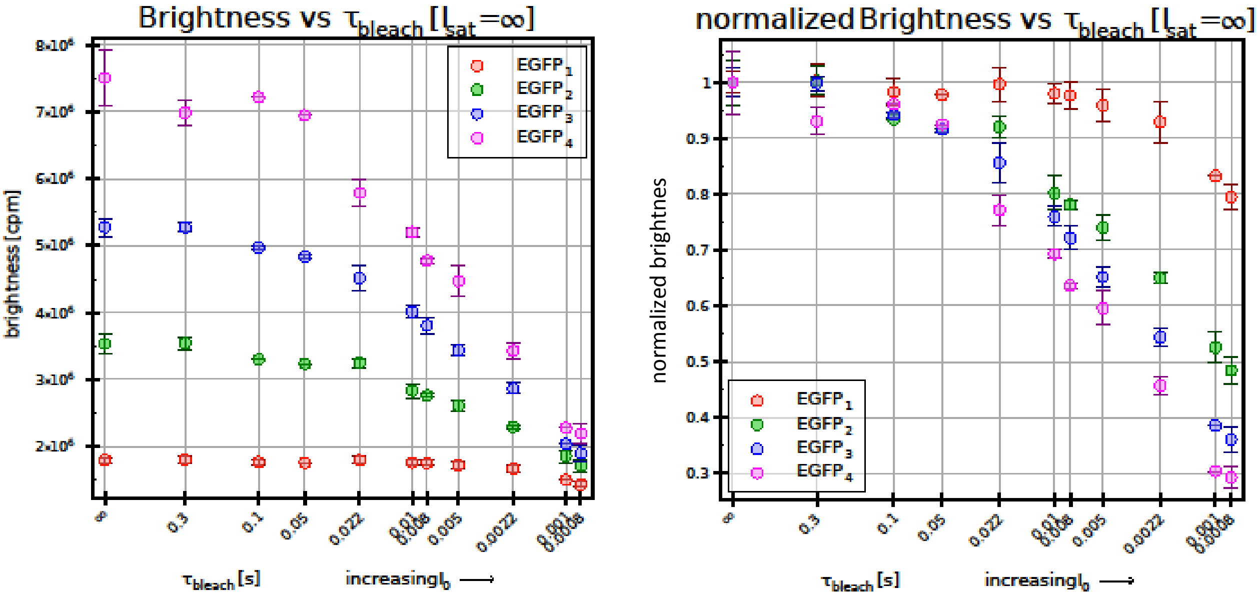

Supplementary Figure S4 (continued)

Simulation of the effect of photobleaching and excitation saturation on the parameters of $EGFP_{1-4}$ oligomers.

e) Apparent brightness (left) and its normalized value (right) vs. the illumination power I_0 relative to the saturating power I_{sat} in the absence of bleaching. **f)** Apparent brightness vs. τ_{bleach}.
